# Expression Profiling of Regulatory and Biosynthetic Genes in Contrastingly Anthocyanin Rich Strawberry (*Fragaria* × *ananassa*) Cultivars Reveals Key Genetic Determinants of Fruit Color

**DOI:** 10.3390/ijms19030656

**Published:** 2018-02-26

**Authors:** Mohammad Rashed Hossain, Hoy-Taek Kim, Ashokraj Shanmugam, Ujjal Kumar Nath, Gayatri Goswami, Jae-Young Song, Jong-In Park, Ill-Sup Nou

**Affiliations:** 1 Department of Horticulture, Suncheon National University, 255 Jungang-ro, Suncheon, Jeonnam 57922, Korea; m.r.hossain@bau.edu.bd (M.R.H.); araj866@gmail.com (A.S.); ujjalnath@gmail.com (U.K.N.); gayatri_bau@yahoo.com (G.G.); jipark@sunchon.ac.kr (J.-I.P.); 2 Department of Genetics and Plant Breeding, Bangladesh Agricultural University, Mymensingh 2202, Bangladesh; 3 University-Industry Cooperation Foundation, Suncheon National University, 255 Jungang-ro, Suncheon, Jeonnam 57922, Korea; 4 Department of Crop Science, Chungbuk National University, Chengju 28644, Korea; novaplant@cbnu.ac.kr

**Keywords:** *Fragaria × ananassa*, anthocyanin, flavonoid, transcriptional regulation, biosynthetic genes

## Abstract

Anthocyanins are the resultant end-point metabolites of phenylapropanoid/flavonoid (F/P) pathway which is regulated at transcriptional level via a series of structural genes. Identifying the key genes and their potential interactions can provide us with the clue for novel points of intervention for improvement of the trait in strawberry. We profiled the expressions of putative regulatory and biosynthetic genes of cultivated strawberry in three developmental and characteristically colored stages of fruits of contrastingly anthocyanin rich cultivars: Tokun, Maehyang and Soelhyang. Besides *FaMYB10,* a well-characterized positive regulator, *FaMYB5*, *FabHLH3* and *FabHLH3-delta* might also act as potential positive regulators, while *FaMYB11*, *FaMYB9*, *FabHLH33* and *FaWD44-1* as potential negative regulators of anthocyanin biosynthesis in these high-anthocyanin cultivars. Among the early BGs, *Fa4CL7*, *FaF3H*, *FaCHI1*, *FaCHI3*, and *FaCHS,* and among the late BGs, *FaDFR4-3*, *FaLDOX*, and *FaUFGT2* showed significantly higher expression in ripe fruits of high anthocyanin cultivars Maehyang and Soelhyang. Multivariate analysis revealed the association of these genes with total anthocyanins. Increasingly higher expressions of the key genes along the pathway indicates the progressive intensification of pathway flux leading to final higher accumulation of anthocyanins. Identification of these key genetic determinants of anthocyanin regulation and biosynthesis in Korean cultivars will be helpful in designing crop improvement programs.

## 1. Introduction

Strawberry (*Fragaria* × *ananassa*) is favored across the globe not only due to its sweet-sour taste, unique flavors and rich nutritional values but also due to its attractive appearance [1]. These qualitative attributes are largely manifested by plant’s inherent metabolic composition [2] where the flavonoid/phenylpropanoid (F/P) pathway plays key roles in determining the characteristic pigments of strawberry fruit [3,4,5]. The pigment is the resultant effect of accumulation of the secondary metabolites—anthocyanins—which play diverse roles ranging from fruit coloration and flavor to fruit ripening [6,7], increased pollination to seed dispersal via attracting pollinators and predators [8], anti-oxidative roles in protecting against UV light [9] and against abiotic and biotic stresses [5]. Besides, anthocyanins also have potential long-term health benefits including the production of health promoting compounds having anti-oxidative [10], anti-mutagenic and anticancer properties [11]. Anthocyanins thus play pivotal roles in consumer preference and marketability of strawberry fruits [12]. All these made anthocyanins and its biosynthetic pathway one of the well-studied pathways in strawberry and other fruits at genetic, biochemical and molecular levels [5,10,13,14] and are therefore the targets of many strawberry breeding programs [3,4]. 

The pathway involves several enzymes that act in two stages, namely, early and late biosynthetic stages, leading to the biosynthesis of different flavonoids such as flavonols, condensed tannins and anthocyanidins which give rise to the characteristic colors in flowers and fruits of many species [15,16]. Early biosynthetic stages start with the catalysis of phenylalanine to yield cinnamic acid and coumaroyl-CoA followed by the synthesis of chalcone which is then isomerized to flavanone by the enzyme chalcone isomerase (CHI) [16]. The enzyme flavanone 3-hydroxylase (F3H) catalyzes flavanones to dihydroflavonols. In late biosynthetic steps, dihydroflavonol reductase (DFR) reduces dihydroflavonols to leucoanthocyanins which in turn is converted to anthocyanidins by leucoanthocyanidin dioxygenase/anthocyanidin synthase (LDOX/ANS). The anthocyanidins are finally glycosylated to anthocyanins via the enzyme uridine diphosphate (UDP)-glucose:flavonoid-*O*-glycosyl-transferase (UFGT) [5,17]. 

These structural genes involved in the biosynthesis of anthocyanins are mainly regulated at transcriptional level via a ternary regulatory *MYB-bHLH-WD40* “*MBW*” protein complex which is formed by highly conserved transcription factors (TFs) *R2R3-MYB* interacting or not with MYC-like basic helix-loop-helix (*bHLH*) proteins and/or with *WD40*-repeat proteins [12,18]. The TFs of the *MBW* complex is identified in many species including *Arabidopsis* [19], grapevine [20], tomato and pepper [21], maize [22], potato [23], apple [24,25], sweet cheery [26,27], etc. The *MBW* complex is unique to plants, varies in monocot vs. dicot species, and not all three members of the complex are needed for anthocyanin biosynthesis in every species [16,17]. For example, in maize (monocot), TFs of *MBW* complex activates the biosynthesis of anthocyanin as a single unit [28]. In Arabidopsis, the early biosynthetic genes can be regulated by an independent *R2R3-MYB* co-activator while late biosynthetic genes are known to be regulated by ternary *MBW* complex [16]. In grape and apple, *MYB-bHLH* complex (lacking *WD40*) regulates anthocynin biosynthesis [25]. In strawberry, *FaMYB10* is reported as the key regulator of anthocyanin biosynthesis [29]. Besides these activators, few TFs may act as negative regulator of the pathway in different species such as *FaMYB1* and *FcMYB1* in strawberry [30,31]; *MdMYB16*, *MdMYB17* and *MdMYB111* in apple [32]; and *AtMYB3*, *AtMYB4* and *AtMYBL2* in Arabidopsis [33].

The combinatorial interactions of the regulatory genes activate or inhibit the expression of individual or set of structural genes (some of which might have pleiotropic effects) that act in a coordinated manner to control the flux of various branches of the pathway which ultimately determines the flavonoid composition in the tissue [3,12,15]. The pathway is also modulated by environmental factors, especially light and temperature, which further influence the qualitative and quantitative composition of anthocyanins [34,35,36]. 

Thus, studying the cultivar specific regulatory, early- and late-biosynthetic genes together will be helpful in identifying the overall association of the genes involved and the key steps of the anthocyanin biosynthetic pathway which can enable us to target specific gene(s) and develop molecular markers for breeding programs for improving pigmentation in strawberry. The current study thus investigates the key genetic determinants of anthocyanin accumulation in three popular contrastingly pigmented strawberry cultivars of Korea by associating the expressions of all regulatory, early- and late-biosynthetic genes with the accumulation of anthocyanins in strawberry fruits based on a multivariate statistics based approach.

## 2. Results

### 2.1. Total Anthocyanin Varied Across Fruit Developmental Stages and Cultivars of Strawberry

**Figure 1:**
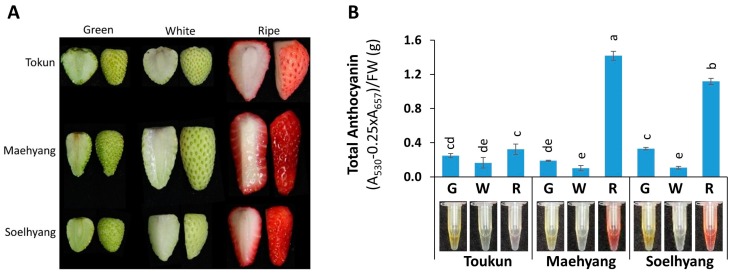
Strawberry (*Fragaria* × *ananassa*) fruits (**A**); and contents of total anthocyanin (**B**) in three developmental stages of fruits, namely, green, white and ripe stages of cultivar, Tokun, Maehyang and Soelhyang are used in this study. Data are presented as mean ± SD (*n* = 3). Fruit developmental stages of the cultivars varied significantly (*p* < 0.01) for total anthocyanin as determined by one-way ANOVA. Statistically significant differences in the total anthocyanin content are indicated by different letters as per Tukey’s pairwise comparisons. G, Green; W, White; R, Ripe.

In general, total anthocyanin contents varied significantly (*p* < 0.01) across the fruit developmental stages and were higher in ripe fruits followed by green and white fruits in all three cultivars (Figure 1B). A generalized decrease in the total anthocyanin contents in white stage; a less pigmented transitional stage between green and ripe stages were observed in all three cultivars. Notable was that the ripe fruits of Maehyang and Soelhyang contained significantly higher amount of total anthocyanin (approximately six- and five-fold higher, respectively, compared to that of the ripe fruits of Tokun) which was apparent from the visual observations of the ripe fruits and the tubes containing anthocyanin extract as well (Figure 1A,B). This clearly indicates Maehyang and Soelhyang as high anthocyanin containing cultivars compared to Tokun based on the content of anthocyanin in ripe fruits in particular.

### 2.2. FaMYB10 Dominated the Anthocyanin Regulatory Complex 

**Figure 2:**
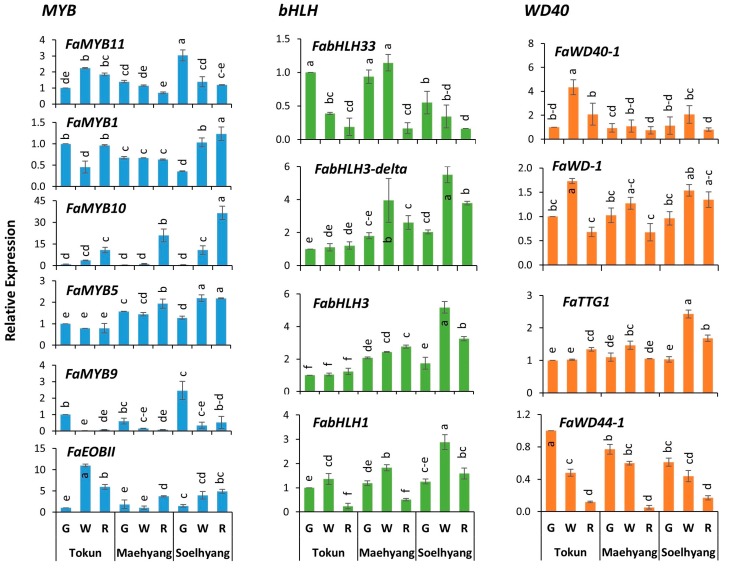
Expression analysis of the regulatory genes involved in the biosynthesis of anthocyanin by quantitative real-time PCR in the three fruit developmental stages, namely, green, white and ripe fruits of *Fragaria* × *ananassa* fruit. Data are presented as mean ± SD (*n* = 3). Fruit developmental stages of the cultivars varied significantly (*p* < 0.01) for relative expressions as determined by one-way ANOVA. Statistically significant differences in the relative expressions are indicated by different letters as per Tukey’s pairwise comparisons. G, Green; W, White; R, Ripe.

Highly variable expressions were obtained for the studied 14 regulatory genes of *MBW* protein complex in the three developmental stages of fruits of three differentially anthocyanin containing cultivars. Among the *MYB* transcription factors (TFs), *FaMYB10*, a well-known *MYB* TF, showed remarkable higher expressions in the ripe fruits of high anthocyanin containing cultivars, Maehyang and Soelhyang (Figure 2). Compared to the expressions of the green fruits of low anthocyanin containing cultivar Tokun, the gene expressions were ~21- and 37-fold higher, respectively (~2- and ~3-fold higher than the ripe fruits of Tokun, respectively). The gene *FaMYB5* showed slightly increased statistically significant expressions in the ripe fruits of Maehyang and Soelhyang compared to the respective green fruits while its expressions remained unchanged in all three stages of the cultivar Tokun. Expression of *FaMYB1* significantly increased only in the ripe fruits of Soelhyang compared to initial developmental stages of fruits while it remained unchanged in the other high anthocyanin containing cultivar Maehyang. Another volatile related *MYB* TF, *FaEOBII* (GENE28435), also showed significant increase in the ripe fruits of Maehyang and Soelhyang but its expression was also increased in the low anthocyanin containing cultivar Tokun.

Among the four *bHLH* genes, *FabHLH3* and *FabHLH3-delta* showed significantly increased expressions in the ripe fruits of Maehyang and Soelhyang compared to that of the green fruits, respectively, while its expression in the fruits of Tokun remained statistically unchanged (Figure 2). Of these, only the *FabHLH3*, however, exhibited significant positive correlation (*p* < 0.05) with *FaMYB10* (Table S1). Among the *WD40* protein coding genes, only the expressions of *FaTTG1* were found to be significantly higher in the white and ripe fruits of Soelhyang while the expression of the other three genes were either decreased or statistically similar in both low and high anthocyanin cultivars. Taken together, it is apparent that *FaMYB10* dominated the regulatory complex with approximately 20- and 36-fold higher expressions, along with the *FabHLH3* and *FabHLH3-delta* genes, which showed approximately 3–4 times higher expression in ripe fruits of Maehyang and Soelhyang compared to that of the green fruits of low anthocyanin cultivar Tokun.

### 2.3. FaMYB11 Is the Potential Negative Regulator of Anthocyanin Biosynthesis

Among the *MYB* TFs, the expressions of *FaMYB11* were decreased with the progressive developmental stages of fruits in high anthocyanin containing cultivars Maehyang and Soelhyang (decreased by approximately two and three folds compared to the respective green fruits, respectively) while its expression was significantly increased in low anthocyanin cultivar Tokun (Figure 2). This contrasting pattern of expression in high and low anthocyanin cultivars makes this gene the potential repressor of anthocyanin biosynthesis. Expressions of another *MYB*, *FaMYB9*, were decreased in both high and low anthocyanin cultivars (approximately 5–7 and 15 times, respectively) compared to the respective green fruits. Among the *bHLH* TFs, expressions of *FabHLH33* were also decreased (by approximately 3–6 folds) significantly in ripe fruits of all three cultivars. Such general decrease in expression (by approximately 4–15 folds) was also observed for *FaWD44-1* in all three cultivars which showed significant negative correlation with *FaMYB10* (*p* < 0.001; Table S1)*,* the key positive regulator of anthocyanin biosynthesis. The expression of *FabHLH1*, *FaWD40*-1 and *FaWD-1* showed an increase in white stage before being decreased in the ripe fruits of all three cultivars (Figure 2); a pattern which needs further investigation to determine their specific roles.

### 2.4. Expression Profiles of Early Biosynthetic Genes

**Figure 3:**
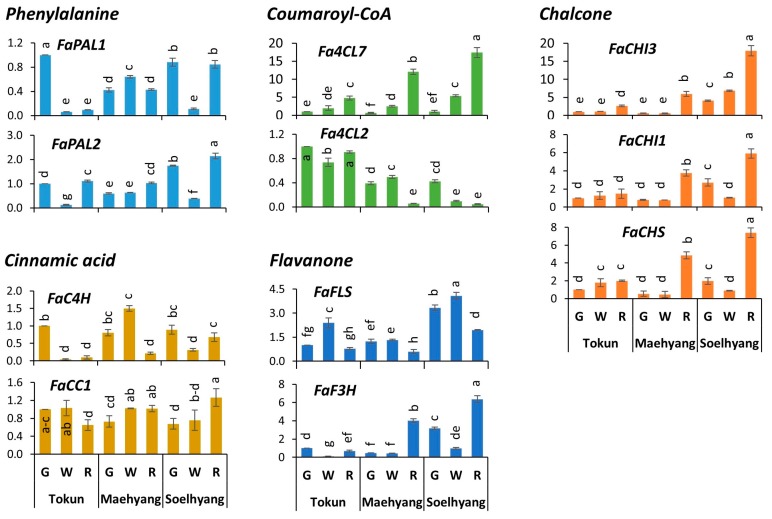
Expression analysis of the genes involved in the early biosynthetic steps of anthocyanin biosynthetic pathway by quantitative real-time PCR in the three fruit developmental stages, namely, green, white and ripe fruits, of *Fragaria* × *ananassa* fruit. Data are presented as mean ± SD (*n* = 3). Fruit developmental stages of the cultivars varied significantly (*p* < 0.01) for relative expressions as determined by one-way ANOVA. Statistically significant differences in the relative expressions are indicated by different letters as per Tukey’s pairwise comparisons. G, Green; W, White; R, Ripe.

Expressions of the structural genes involved in the major metabolic steps of the anthocyanin biosynthesis were studied and significant variations were obtained across the fruit developmental stages and across contrastingly anthocyanin rich cultivars. Among the genes involved in early biosynthetic steps (phenylalanine to flavanone), the genes of the first two steps, namely, phenylalanine and cinnamic acid, did not show any notable higher expressions except phenylalanine ammonia lyase gene *FaPAL2,* which showed a two-fold higher expression in ripe fruits of Soelhyang (compared to green fruits of Tokun) only. However, the expressions of phenylalanine gene *FaPAL1* and cinnamate-4-hydroxylase gene *FaC4H* were significantly decreased in the ripe fruits of low anthocyanin cultivar Tokun (compared to green fruits) while their expressions remained somewhat unchanged in the high anthocyanin cultivars, Maehyang and Soelhyang (Figure 3).

The expression of Coumaroyl-CoA ligase gene *Fa4CL7* were significantly increased from green to ripe fruits in all three cultivars. However, the increase was much higher in the high anthocyanin cultivars Maehyang (~12 folds) and Soelhyang (~17 folds) compared to that of low anthocyanin cultivar Tokun (only ~5 folds). This indicates its potential role in overall anthocyanin biosynthesis. The expression of the other Coumaroyl-CoA ligase gene, *Fa4CL2*, showed ~7- and ~9-fold decreases in the ripe fruits compared to green fruits of high anthocyanin cultivars Maehyang and Soelhyang, respectively, but remained unchanged in low anthocyanin cultivar Tokun (Figure 3). 

Among the genes involved in Chalcone synthesis, both the Chalcon Isomerase genes, *FaCHI1* and *FaCHI3*, and Chalcon Synthase gene, *FaCHS*, showed much higher expressions in the ripe fruits of high anthocyanin cultivars Maehyang and Soelhyang compared to that of the low anthocyanin cultivar Tokun, indicating the importance of this step (and the genes involved) in overall anthocyanin biosynthesis. For example, *FaCHI3* was ~10- and ~4-fold higher expressed in Maehyang and Soelhyang, respectively, while it was only expressed ~2 folds in Tokun.

Among the genes involved in Flavanone biosynthesis, flavanone 3 hydroxylase (*FaF3H*) was expressed by ~8 and ~2 folds in the ripe fruits of Maehyang and Soelhyang, respectively, compared to respective green fruits, while its expression did not show such increase in Tokun ripe fruits. Flavonol synthase on the other hand showed general decrease in the ripe fruits of all three cultivars.

### 2.5. Key Genes of the Late Biosynthetic Steps

**Figure 4:**
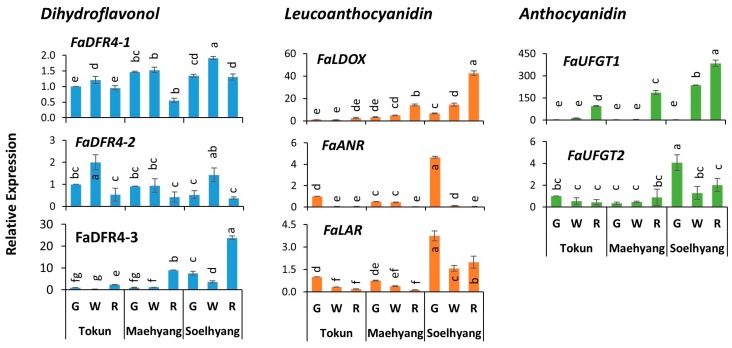
Expression analysis of the genes involved in the late biosynthetic steps of anthocyanin biosynthetic pathway by quantitative real-time PCR in the three fruit developmental stages, namely, green, white and ripe fruits, of *Fragaria* × *ananassa* fruit. Data are presented as mean ± SD (*n* = 3). Fruit developmental stages of the cultivars varied significantly (*p* < 0.01) for relative expressions as determined by one-way ANOVA. Statistically significant differences in the relative expressions are indicated by different letters as per Tukey’s pairwise comparisons. G, Green; W, White; R, Ripe.

Higher expressions were observed for the genes involved in each of the late biosynthetic steps (Figure 4). Among the dihydroflavanol reductase genes, *FaDFR4-3* showed increasing trend of expression from green to ripened stages of fruits in all three cultivars. However, the expressions were much higher in the ripe fruits of high anthocyanin cultivars, Maehyang and Soelhyang (~9 and ~24 folds, respectively) compared to only 2.29-fold increase in the low anthocyanin cultivar Tokun (Figure 4). The expressions of the other two genes, *FaDFR4-1* and *FaDFR4*-2, did not show such contrastingly increasing patterns of expression between high and low anthocyanin cultivars. For these two genes, the expressions were similar in green and ripe fruits, but, interestingly, a general increase in the intermediate white stage is observed for each of the three cultivars.

Among the genes involved in the leucoanthocyanidin step, leucoanthocyanidin dioxygenase *FaLDOX* showed increasing pattern of expression from green to ripened stages of fruits in all three cultivars. In the ripe fruits of high anthocyanin cultivars, Maehyang and Soelhyang, the gene showed ~14- and ~42-fold higher expressions compared to the green fruits of low anthocyanin cultivar Tokun (four and six folds compared to respective green fruits) (Figure 4). The other two genes, namely, Anthocyanidin reductase (*FaANR*) and Leucoanthocyanidin reductase (*FaLAR*), showed decreasing patterns of expression in all three cultivars. Anthocyanidin reductase (*FaANR*), in particular, was almost not expressed in the ripe fruits, while the green fruits showed significantly high expressions indicating a possible repressing role played by this genes in overall anthocyanin biosynthesis pathway.

The most striking increasing pattern of expression was obtained for the uridine diphosphate-glucose:flavonoid 3-*O*-glucosyltransferase gene, *FaUFGT1*, involved in the very final step of anthocyanin biosynthetic pathway, showing around 123- and 313-fold higher expressions (compared to the respective green fruits) in the ripe fruits of high anthocyanin cultivars, Maehyang and Soelhyang (Figure 4).

### 2.6. Late Biosynthetic Genes Show Comparatively Higher Expression

A general notion is observed that, compared to the expressions of the early biosynthetic genes, late biosynthetic genes showed higher expressions in the ripe fruits of high anthocyanin cultivars, Maehyang and Soelhyang. For example, the highest relative expressions observed for early biosynthetic genes were 17.90 (*FaCHI3*); 17.41 (*Fa4CL7*) in the ripe fruits of high anthocyanin cultivar Soelhyang compared to the green fruits of low anthocyanin cultivar Tokun (Figure 3). Few of the late biosynthetic genes showed much higher expressions such as *FaDFR4-3* (23.84), *FaLDOX* (42.48) and *FaUFGT1* (384.29) in the ripe fruits of high anthocyanin cultivar Soelhyang (compared to the green fruits of low anthocyanin cultivar Tokun) (Figure 4). This probably indicates much greater role of the final steps of the anthocyanin biosynthesis pathways (and the genes involved in those steps) in final accumulation of anthocyanin in ripe fruits which give rise to the characteristic color in ripe fruits. 

### 2.7. Association between Contents of Total Anthocyanin and Expressions of Related Regulatory and Biosynthetic Genes

**Figure 5:**
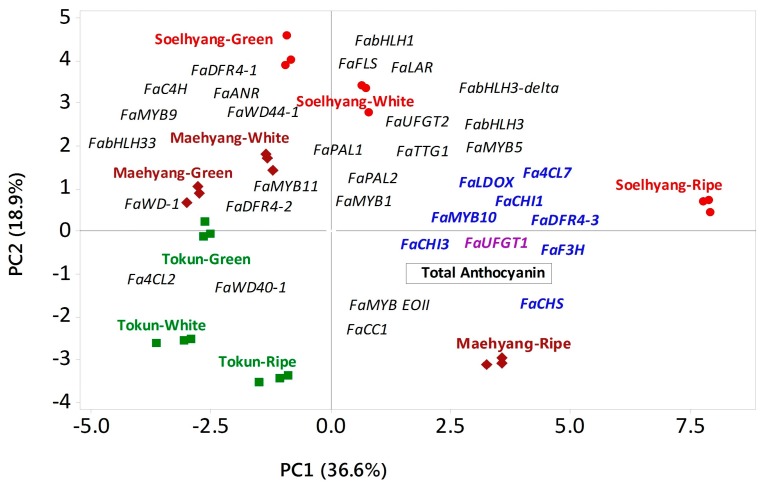
Biplot showing the association between the regulatory and biosynthetic genes with the total anthocyanin in three fruit developmental stages, namely, green, white and ripe fruits, of *Fragaria × ananassa* fruit as determined by Principal Component Analysis (PCA). The three varieties are shown in three different shapes and colors as positioned by their respective mean PC scores in PCA biplot. The total anthocyanin (shown in rectangular box) and the genes represent coefficients between PC1 and PC2. The most contributing genes in accumulating higher anthocyanin in ripe fruits of cultivars, Maehyang and Soelhyang, are shown by bold letters. PC, Principal Component.

Principal Component Analysis (PCA) of the contents of total anthocyanin, the expressions of regulatory and biosynthetic genes in three developmental stages of fruit ripening in contrasting cultivars extracted six principal components (PCs) having eigenvalue greater than unity (data not shown). The first three PCs explained 73.5% of the total variation in the entire datasets. PC1 accounted for 36.6% of the total variation which is mainly manifested by the higher positive coefficients of total anthocyanins (0.23), *MYB10* (0.26), *Fa4CL7* (0.27), *FaF3H* (0.26), *FaCHI3* (0.27), *FaCHI1* (0.25), *FaCHS* (0.26), *FaDFR3* (0.26) and *FaUFGT1* (0.26) versus higher negative coefficients of *FabHLH33* (−0.173), *FaWD40*-1 (−0.104), *FaWD44-1* (−0.176), *Fa4CL2* (−0.204), *FaDFR-2* (−0.136) and *MYB11* (−0.080) (Table 1 and Figure 5). PC1 clearly distinguished the key highly expressed genes from the rest and the anthocyanin rich ripe fruits of Maehyang and Soelhyang from the other less anthocyanin containing samples as evident by their mean PC scores in opposite direction in these contrasting samples and differential positioning in PCA biplot (Table 1 and Figure 5). The highly expressed genes were plotted along the total anthocyanin content and ripe fruit samples of high anthocyanin cultivars, Maehyang and Soelhyang as shown in PCA biplot (Figure 5). This is also corroborated by the higher significant positive correlations between total anthocyanin and these genes, as observed from the Pearson’s correlation analysis (Table S1).

## 3. Discussion

Anthocyanins are plant’s secondary metabolites that render the attractive pigmentation and characteristic flavor to many fruits along with potential health benefits to human. This study attempted to identify the key regulatory and structural genes and their association with total anthocyanins in contrastingly pigmented strawberry cultivars. 

### 3.1. Positive Regulators of Anthocyanin Biosynthesis in Korean Strawberry Cultivars

Expression analysis of the 14 selected regulatory genes in the fruits of contrasting cultivars identified *FaMYB10* as the most highly expressed and *FaMYB5, FabHLH3* and *FabHLH3-delta* as somewhat (~3–4 folds) highly expressed TFs in high anthocyanin containing fruits (Figure 2). No such definitive pattern of increase was observed for any of the four *WD40* repeat proteins investigated. This indicates the regulatory complex of the strawberry is dominated by *R2R3-MYB* TF, and *FaMYB10* with little influence of *bHLH* counterparts and that the regulatory complex in our studied materials lacks *WD40* protein. Orthologs of *MYB10* were identified to be involved in the biosynthesis of anthocyanins during ripening of more than 20 different fruits of *Rosaceae* [25,29]. In strawberry, this gene was found to be expressed in fruit receptacles particularly during ripened to senescent stages compared to earlier stages of fruit development, with negligible expressions being observed in all vegetative tissues (e.g., roots, leaves, crowns, and runners) and in fruit achenes [37].

The composition of the members of this *MBW* complex is known to vary across species [3,16,29]. Among the *MBW* complex proteins, *bHLH* and WD proteins have broader and overlapping regulatory targets, while the regulatory targets of *MYB*s are known to be specific, as evident by the involvement of different *MYB*s for different biosynthetic steps [3,12,16]. For apple and grape, only *MYB* and *bHLH* proteins (lacking *WD40*) regulate the biosynthesis of anthocyanin [24,25]. In Arabidopsis, only an independent *R2R3-MYB* TF lacking both *bHLH* and *WD40* counterparts regulates the early biosynthetic genes while the late biosynthetic genes are regulated by *MBW* complex proteins [16]. The role of *bHLH* was extensively investigated in rosaceous species and it was observed that the R2R3 domain of *MYB10*s of 20 rosaceous contains several key motifs that indicates its association with suitable *bHLH* counterpart [29]. Many of the rosaceous *MYB*s including strawberry, apple and cherry were observed to promote *DFR* activity when transiently co-infiltrated with either apple or Arabidopsis *bHLH* genes. In both wild and cultivated strawberry, the Arabidopsis *bHLH* genes, *AtbHLH2* and *AtbHLH42* significantly increased the *DFR* activity [29]. The promoters of *DFR* and *UFGT* of wild strawberry was significantly increased by *FvMYB10* and *FvbHLH33* as observed by dual luciferase assay in *Nicotiana benthamiana* [38].

Besides the *FaMYB10*, significant higher expressions (2.4–2.7 folds) were observed for *FaMYB5* in the ripe fruits of high anthocyanin containing cultivars. *FaMYB5* also showed significant positive correlation (*p* < 0.01) with *FaMYB10* which may indicate the existence of regulatory role of *FaMYB5* (Table S1) at least in these high-anthocyanin cultivars. This gene (*FaMYB5*) was hypothesized to have contrasting positive roles during fruit developmental stages which regulates proanthocyanidin biosynthesis during early- and anthocyanin biosynthesis during late (ripening)-developmental stages [2]. The two *bHLH* positive regulators, *FabHLH3* and *FabHLH3-delta*, in this study were interestingly found to be the negative regulator of proanthocyanidin biosynthesis in strawberry [2]. The significant and contrastingly higher expressions of *FaMYB5*, *FabHLH3* and *FabHLH3-delta,* besides *FaMYB10*, in the ripe fruits of high anthocyanin containing cultivars and the positive correlation of these genes (which are positively correlated between themselves as well) with total anthocyanin and key structural genes such as *FaDFR4-3, FaLDOX* and *FaUFGT1* indicate their positive contribution in anthocyanin biosynthesis in these cultivars. 

### 3.2. Potential Repressors of Anthocyanin Biosynthesis

The contrasting patterns of expressions in high and low anthocyanin cultivars indicate *FaMYB1*1 as potential negative regulator of anthocyanin biosynthesis in the studied genotypes. Besides, expression of *FaMYB9* decreased sharply in both high (~5–7 times) and low (~15 times) anthocyanin cultivars and the fact that its expression is more decreased in low anthocyanin cultivar indicates its potential role in repressing anthocyanin biosynthesis also (Figure 2). Several negative regulators of anthocyanin biosynthesis is identified in different species such as *MdMYB16*, *MdMYB17* and *MdMYB111* in apple [32]; and *AtMYB3*, *AtMYB4* and *AtMYBL2* in Arabidopsis [33]. To our best knowledge, no previous report of *FaMYB11* and *FaMYB9* as negative regulator of anthocyanin biosynthesis in *Fragaria* × *ananassa* is available indicating the need for further studies to confirm the exact roles of these genes in strawberry. 

*FaMYB1* was proposed as a transcriptional repressor of anthocyanin biosynthesis as overexpressing this gene in tobacco has shown to reduce pigmentation via reduced activity of *ANS* and *UFGT* genes (of lower end of F/P pathway that leads to the final accumulation of anthocyanin) [30]. However, the gene was found to be highly expressed in red-ripe strawberry fruits [30]. However, its ortholog, *FcMYB1*, has shown to be highly expressed in white Chilean strawberry (*Fragaria chiloensis*) compared to that in the red fruits of *Fragaria* × *ananassa* cv. camarosa whose silencing in white Chilean strawberry has increased the level of *ANS* and reduced those of *ANR* and *LAR* reverting the pathway to produce partially red phenotype [31]. In *F. vesca*, the repressor *MYB1R* was significantly up-regulated in yellow fruits compared to red fruits [39]. We observed differential expression of this genes along green to ripe stages: unaffected in one (Maehyang) and increased in another (Soelhyang) high anthocyanin cultivar, while its expression in the ripe fruits of low anthocyanin cultivar did not change significantly during fruit ripening. This indicates the differential role of this gene in our tested cultivars. 

Another *bHLH* TF, *bHLH33*, when co-transformed with *MYB10*, has shown strong activation effect on the apple *MYB10* promoter [25] which, however, did not show such activation of the *FvMYB10* promoter [38]. However, co-expression of *FvbHLH33* with *FvMYB10* had shown to strongly activate *AtDFR*, *FvDFR*, and *FvUFGT* promoters in *Nicotiana benthamiana* plants [38] indicating its positive role in activating key anthocyanin pathway genes. We, however, observed a generalized decrease in transcript level of *FabHLH33* across fruit developmental stages in all three cultivars. Similar decreasing trend was also observed for *FaWD44-1*. Thus, besides the contrasting expressions of *FaMYB11* in high and low anthocyanin cultivars, which makes it an obvious potential negative regulator, the generalized decreasing trend of expressions of *FaMYB9, FabHLH33* and *FaWD44-1* along fruit developmental stages in both high and low anthocyanin cultivars also indicates the existence of potential negative influence of these genes on anthocyanin biosynthesis, at least in the studied strawberry cultivars. This is further evident from the fact that these four genes are negatively correlated with the key positive regulator *FaMYB10* and with total anthocyanin.

### 3.3. Anthocyanin and Proanthocyaninid Might Share Few Contrasting Regulatory Genes

Discussing the role of regulatory genes of anthocyanin and proanthocyanidin biosynthesis, it became apparent that some regulatory genes may have contrasting roles in these two processes. For example, besides *FaMYB10*, we observed positive roles of *FaMYB5*, *FabHLH3* and *FabHLH3-delta* in the biosynthesis of anthocyanin in our strawberry genotypes (Figure 2). Among these, *FaMYB5* and *FabHLH3-delta* were reported as negative regulators of proanthocyanidin biosynthesis in strawberry [2]. While *FaMYB11* and *FaMYB9* appeared as potential negative regulators of anthocyanin biosynthesis in our study, these two genes were found to act as positive regulator of proanthocyanidin biosynthesis [2]. *FaMYB9*/*FaMYB11* along with *FabHLH3* and *FaTTG1* form a ternary complex which is shown to upregulate leucoanthocyanidin- and anthocyanidin-reductase (*LAR* and *ANR*, respectively) causing an increase in proanthocyanidin contents [2]. Silencing of one of these PA biosynthesis enzymes, *ANR* has shown to revert the F/P pathway to produce anthocyanin instead of PAs during early developmental stages of strawberry fruits [40]. Contrastingly, overexpression of the Arabidopsis orthologs of these genes (i.e., *AtTT2*, *AtTT8* and *AtTTG1*, respectively) has shown to decrease anthocyanin and increase PA in strawberry further indicating their contrasting roles in the biosynthesis of two final steps (i.e., biosynthesis of anthocyanins and proanthocyanidins) of F/P pathway. Besides, few genes have shown common effect on accumulation of both anthocyanin and proanthocyanidin such as *FabHLH3* and *FaTTG1* as evident by their positive roles in PA biosynthesis [2] and higher expression in our high anthocyanin containing ripe fruits.

### 3.4. Key Structural Genes and Their Association with Total Anthocyanin and Regulatory Genes

Based on our comparative univariate expression analysis considering the expressions of green fruits of low anthocyanin cultivar, Tokun as control, we identified *FaPAL2*, *FaCC1*, *Fa4CL7*, *FaCHI1*, *FaCHI3*, *FaCHS* and *FaF3H* as the key early- and *FaDFR4-3*, *FaLDOX* and *FaUFGT1* as the key late-biosynthetic genes (Figure 2, Figure 3 and Figure 4). We employed multivariate analytical approach on the entire datasets to identify and visualize the overall association of these genes with total anthocyanins which may have arisen from the patterns of changes in the expressions of the genes and the contents of total anthocyanin in the three progressive fruit developmental stages of high- and low-anthocyanin cultivars. Positioning of the highly expressed genes along with total anthocyanin content and high anthocyanin containing ripe fruit samples of Maehyang and Soelhyang in close vicinity in PCA biplot indicates the close association between these factors which can be translated to the fact that the activity of these genes in ripe fruits of Maehyang and Soelhyang leads to the higher accumulation of anthocyanin. With total anthocyanin and *FaMYB10*, the key regulator, only these structural genes have shown higher significant positive correlations which further corroborates the association between these genes and total anthocyanin. A previous study, however, reported no determining role of 4CL in strawberry fruit pigmentation [31], whereas we observed 17.8- and 16.2-fold higher expression of this gene in the ripe fruits of our high anthocyanin containing cultivars compared to the respective green fruits. 

The relationship between the key regulatory gene, *FaMYB10* with other structural genes was previously demonstrated by several transcriptomic, over-expression and gene silencing studies [7,10,37,38,39]. For example, over-expression of *FaMYB10* had increased anthocyanin contents in roots, leaves and fruits of *Fragaria* × *ananassa* [29] and silencing of this genes had downregulated both early- and late-biosynthetic genes of F/P pathway that includes *PAL, C4H, F3H, 4CL, CHS, CHI, DFR* and *UFGT*, etc. [37]. A similar set of structural genes were reported in several other species as well such as in grapevine [17], pear [41], apple [42], potato tuber [43], etc. A little contrast is observed in our correlation based analysis as the gene *FaC4H* was found to be negatively correlated (statistically non-significant) with both total anthocyanin and *FaMYB10* (Table S1). Furthermore, silencing of *FaMYB10* (and also *FvMYB10*) did not show any significant effect on the expression of *LDOX* (or *ANS*) [38]; which prompted to speculate different regulatory mechanism for this gene manifested by another *MYB* transcription regulator *FaMYB5* (whose expression is not regulated by *FaMYB10*) [37]. We observed high significant positive correlation of *FaLDOX* with both *FaMYB10* and *FaMYB5* (with the latter two showing significant positive correlation between themselves too) which indicates the existence of regulatory roles of these two TFs. It is notable to mention in this regard that besides *FaLDOX*; *FaMYB5* also showed significant positive correlations with all the key structural genes to which *FaMYB10* too showed positive correlation (Table S1). Our results with *FaMYB10* seemed to be consistent with the findings that stable over-expression of its *F. vesca* counterpart, *FvMYB10* had increased the expression of *LDOX* [38] as opposed to the previous speculation of having different roles in regulating *LDOX* transcription. As with the specific role of *FaMYB5*, which was contrastingly found to act as a negative regulator of proanthocyanidin biosynthesis in *F. ananassa* [2], further functional investigation is necessary.

### 3.5. Progressive Intensification of Pathway Flux May Lead to Higher Anthocyanin Accumulation

Anthocyanin biosynthesis is a complex multi-enzymatic process requiring the coordinated interaction and systemic expressions of many genes via a highly regulated mechanism within the limits of developmental stages and environmental cues that control the pathway flux across the branch points leading to the final accumulation of the end products [3,5,10,12,16]. A combined study of anthocyanin biosynthesis focusing on the genetic, developmental and environmental influences revealed that expressions of genes, activities of enzymes and levels of flavonoids, all follow a clear developmental pattern in strawberry [14]. An overview on the highly expressed genes of each of the pathway nodes in our study makes it apparent that late biosynthetic genes are comparatively highly expressed compared to the early biosynthetic genes and a general increasing trend of gene expressions (starting from *FaPAL1* having a maximum of 2.14-fold expression through to *FaUFGT2* having a maximum of ~384-fold expression, with *FaCHS* and *FaF3H* causing little fluctuation in this trend) is observed along the progress of the pathway which probably indicates the progressive intensification of the metabolic flux leading to the final accumulation of anthocyanin (Figure S1). Early biosynthetic genes (such as *CHS, CHI*, *F3H*, etc.) are known to catalyze the production of flavonols whereas the late biosynthetic genes (*DFR, LDOX/ANS* and *UFGTs*) are involved in biosynthesis of anthocyanin [44,45]. The existence of different sets of regulatory gene(s) as proposed for the early and late biosynthetic steps [44] may have a role in this differential expression of the early- and late-biosynthetic genes.

## 4. Methods

### 4.1. Plant Materials

Three strawberry (*Fragaria* × *ananassa*) cultivars, namely, Maehyang, Seolhyang and Tokun (also known as “Toukun”) were grown in large rectangular pots using nursery soil mix under standard growth and nutritional conditions in the glass house research facility of Suncheon National University, South Korea. Fruits of three developmental stages, namely, green, white and ripe stages were harvested and were immediately flash frozen in liquid nitrogen before storing at −80 °C for the subsequent quantification of total anthocyanin and extraction of total RNA.

### 4.2. Extraction and Photometric Determination of Anthocyanin

Total anthocyanins were extracted from the liquid nitrogen frozen fruit samples following the procedures described by [35] with minor modifications. In short, anthocyanin were extracted in 1 mL of acidic methanol (1% HCl, *w*/*v*) by incubating 100 mg of finely grounded fruit tissue powder at room temperature for 18 h in dark followed by clearing up the extract by centrifugation at 14,000 rpm for 10 min. Total anthocyanins were quantified based on the absorption of the extracts using the equation: *Q_Anthocyanins_* = (*A_530_* − 0.25 × *A_657_*) × *FW*^−1^, where *Q*_Anthocyanins_ is the amount of total anthocyanins; *A*_530_ and *A*_657_ are the absorptions at 530 nm and 657 nm, respectively; and *FW* is the weight of plant materials (g). Anthocyanins were quantified as triplicates of three independent biological replicates. 

### 4.3. Selection of Anthocyanin Related Genes in Fragaria × ananassa

**Figure 6:**
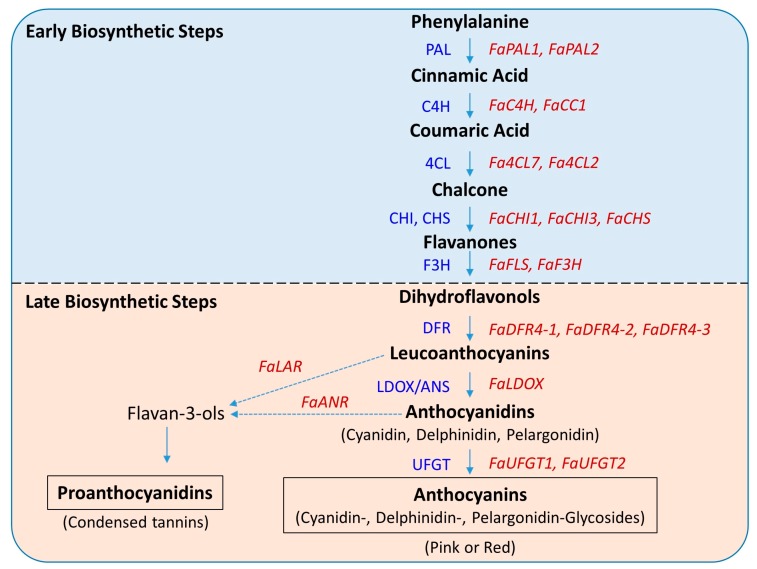
Simplified schematic representation of phenylapropanoid/flavonoid biosynthesis pathway leading to the accumulation of anthocyanins in *Fragaria × ananassa.* Enzymes involved in each of the intermediate steps are shown by blue text in the left side of the arrow and the genes whose expressions were studied were shown by red text in the right side of the arrow. Key flavonoid end products are shown in boxes. Dotted arrows indicate the points where the pathway diverts from biosynthesis of anthocyanin to proanthocyanidin. Details of the genes involved in each of the steps are shown in Tables 2 and S3.

The genes involved in each step of the anthocyanin biosynthesis pathway were first manually searched using step-specific key words (such as “DFR”, “Dihydroflavanol”, etc. for identifying the *DFR* genes) from the annotated version of the whole genome of wild strawberry, *Fragaria vesca* (“Fvesca_V1.0_genemark_hybrid annotation” file, available from the Genome Database of Rosaceae in https://www.rosaceae.org/) (Table S2). Among these genes, the important regulatory, early- and late-biosynthetic genes whose expressions were to be studied in cultivated strawberry, *Fragaria × ananassa* were then selected based on their prior reports in previous studies on *Fragaria vesca* and/or *Fragaria × ananassa*. The *F. vesca* (wild strawberry) sequences of selected genes (from Table S2) were then used to identify corresponding *Fragaria* *× ananassa* (cultivated strawberry) sequences (including the isoforms) by using the “BLAST” tool against the *Fragaria* *× ananassa* draft genome (FANhybrid_r1.2_cds, available from the Strawberry garden database—http://strawberry-garden.kazusa.or.jp/). The list of the selected genes is shown in Table 2; the stepwise biosynthetic genes are marked in anthocyanin biosynthesis pathway in Figure 6; and the complete information are given in Table S3.

### 4.4. RNA Extraction and Expression Analysis via Real-Time qRT-PCR

Total RNA was isolated from the liquid nitrogen frozen fruit samples of three fruit developmental stages, namely, green, white and ripening stages, using RNeasy mini kit (Qiagen, Inc., Redwood, CA, USA) based on manufacturer’s instruction. The samples were treated with RNAse free DNase (Qiagen) to remove any genomic DNA contamination. Conversion of total RNA into cDNA was carried out using Superscript-III^®^ First-strand Synthesis Supermix kit (Invitrogen, Carlsbad, CA, USA). The purity and concentration were determined spectrophotometrically using Nanodrop-2000 (Nanodrop Technologies, Wilmington, DE, USA). 

The qRT-PCR based expression profiling of the genes were carried out using “Roche-Light Cycler^®^ 96 system” (Roche Diagnostics, Pleasanton, CA, USA). The gene-specific primers were designed for each of the genes using primer3plus (http://primer3plus.com/cgi-bin/dev/primer3plus.cgi) (Table S4). For each reverse transcription reaction, a total volume of 20 μL was prepared containing 10 μL of 2× qPCRBIO SyGreen Mix (PCR Biosystems, London, UK), 1 μL of each of the forward and reverse primers (10 pmoles) and 60 ng/μL of cDNA as template. The qRT-PCR was carried out with denaturation at 95 °C for 10 min, and 45 cycles of amplification with denaturation at 95 °C (20 s), annealing at 55 °C (20 s) and elongation at 72 °C (25 s). Each of the three biological replicates were tested in three technical replicates. Primer specificity was checked by single melting peak. Relative expression for each gene was quantified following Livak’s comparative 2^−ΔΔ*C*t^ method using a Light Cycler^®^ 96 Instrument (Roche Diagnostics, Indianapolis, IN, USA.) [46]. *FaRIB413* was used as the reference gene for expression analysis [47]. Relative expressions for each of the genes were calculated relative to the expression of the respective genes in the green fruit of cultivar, Tokun (control) which was assigned an arbitrary value equal to unity.

### 4.5. Statistical Analysis

Total anthocyanin contents and gene expressions were analyzed by one-way ANOVA and statistically significant differences were analyzed by Tukey’s pairwise comparisons. Data are presented as the average of 3 replicates with error bar indicating standard deviation. The data were standardized (mean subtracted from the variable and then divided by the standard deviation) prior to perform Principal component analysis (PCA). The correlation between the genes and total anthocyanin were measured by Pearson correlation analysis and tested for statistical significance. All statistical analysis was conducted using Minitab v. 17 statistical packages (Minitab Inc., State College, PA, USA).

## 5. Conclusions

This study identified the potential regulators involved in the biosynthesis of anthocyanins in contrastingly anthocyanin rich strawberry cultivars. Multivariate statistics based analytical approaches helped to gain a holistic scenario of overall associations of the genetic determinants involved in the entire process. The results are discussed within the wider context of existing body of knowledge that lead to the identification of few genes having important and/or deferential roles besides the previously known genes. Functional validation of these genes will further widen our understanding of the mechanisms involved. Identification of the key genes will thus be helpful in channeling future efforts towards developing better varieties with improved anthocyanin related traits via breeding and biotechnological means.

## Figures and Tables

**Table 1:** Component loadings and mean PC scores showing the association between total anthocyanin and expressions of regulatory and biosynthetic genes as determined by Principal Component Analysis. PC, Principal Component; p, statistical significance; SD, Standard Deviation.

Variable		PC1	PC2	PC3
**Regulatory Genes**				
*MYB*	*FaMYB11*	−0.080	0.128	−0.115
	*FaMYB1*	0.132	−0.020	0.139
	*FaMYB10*	0.260	−0.094	0.044
	*FaMYB5*	0.205	0.146	0.131
	*FaMYB9*	−0.012	0.253	−0.271
	*FaEOBII*	0.011	−0.216	0.151
*bHLH*	*FabHLH33*	−0.173	0.142	−0.043
	*FabHLH3-delta*	0.128	0.192	0.232
	*FabHLH3*	0.148	0.170	0.238
	*FabHLH1*	0.018	0.271	0.267
*WD40*	*FaWD40-1*	−0.104	−0.110	0.166
	*FaWD-1*	−0.033	0.068	0.239
	*FaTTG1*	0.108	0.154	0.285
	*FaWD44-1*	−0.176	0.198	−0.066
**Early Biosynthetic Genes**				
Phenylalanine	*FaPAL1*	0.064	0.171	−0.264
	*FaPAL2*	0.189	0.078	−0.254
Cinnamic acid	*FaC4H*	−0.047	0.228	−0.113
	*FaCC1*	0.143	−0.059	0.026
Coumaroyl CoA	*Fa4CL7*	0.268	−0.094	0.036
	*Fa4CL2*	−0.204	−0.116	−0.104
Chalcone	*FaCHI3*	0.266	0.051	0.022
	*FaCHI1*	0.254	−0.014	−0.121
	*FaCHS*	0.256	−0.100	−0.074
Flavanone	*FaFLS*	0.018	0.273	0.134
	*FaF3H*	0.258	0.028	−0.137
**Late Biosynthetic Genes**				
Dihydroflavonol	*FaDFR4-1*	−0.035	0.294	0.222
	*FaDFR4-2*	−0.136	0.014	0.216
	*FaDFR4-3*	0.264	0.042	−0.076
Leucoanthocyanidin	*FaLDOX*	0.266	0.059	0.037
	*FaANR*	−0.049	0.233	−0.265
	*FaLAR*	0.074	0.300	−0.157
Anthocyanidin	*FaUFGT1*	0.260	−0.015	0.126
	*FaUFGT2*	0.082	0.245	−0.192
Total anthocyanin		0.229	−0.133	−0.110
Eigenvalue		12.809	6.630	6.280
% variation explained		36.6	18.9	17.9
P (Genotype × Fruit developmental stage)		<0.001	<0.001	<0.001
**Genotype × Fruit Developmental Stage**		**Mean PC Scores ± SD**		
Tokun	Green	−2.75 ± 0.01 g	−0.06 ± 0.01 d	−2.08±0.01 e
	White	−3.15 ± 0.37 g	−2.48 ± 0.02 e	1.87±0.10 b
	Ripe	−1.23 ± 0.14 e	−3.51 ± 0.08 f	−0.45±0.12 cd
Maehyang	Green	−2.64 ± 0.17 fg	0.55 ± 0.26 d	0.04±0.13 c
	White	−2.18 ± 0.30 f	1.54 ± 0.02 c	1.11±0.16 b
	Ripe	3.57 ± 0.06 b	−3.44 ± 0.04 f	−1.10±0.02 de
Soelhyang	Green	−0.66 ± 0.19 d	3.94 ± 0.55 a	−4.26±0.41 f
	White	0.89 ± 0.13 c	2.88 ± 0.30 b	5.01±0.67 a
	Ripe	8.14 ± 0.02 a	0.588 ± 0.36 d	−0.14±0.64 cd

*PAL*, Phenylalanine ammonia lyase; *C4H*, cinnamate-4-hydroxylase; *4CL*, 4-coumaroyl-CoA-ligase; *CHS*, Chalcone synthase; *CHI*, Chalcone isomerase; *F3H*, Flavanone 3 hydroxylase; *FLS*, Flavonol synthase; *DFR*, Dihydroflavanol reductase; *LAR*, Leucoanthocyanidin reductase; *ANR*, Anthocyanidin reductase; *LDOX*, leucoanthocyanidin dioxygenase; *UFGT*, uridine diphosphate-glucose:flavonoid 3-*O*-glucosyltransferase. Statistically significant differences in the relative expressions are indicated by different letters as per Tukey’s pairwise comparisons.

**Table 2:** List of the genes investigated for their involvement in the regulation and early- and late-biosynthesis of anthocyanin in *Fragaria* × *ananassa*. The genes were first manually mined from *F. vesca* (Fvesca_V1.0_genemark_hybrid annotation) genome (Table S2) and then the corresponding sequences of the selected genes were obtained by blasting against *Fragaria × ananassa* genome (FANhybrid_r1.2_cds) available from the Strawberry garden database (http://strawberry-garden.kazusa.or.jp/).

Gene	Gene ID in *Fragaria* × *ananassa* (FANhybrid_r1.2_cds)	CDS (bp)	Annotation	Best Match of *F. vesca* Gene	*e*-Value
**Regulatory Genes**					
***MYB***					
*FaMYB11*	FANhyb_icon00023020_a.1.g00001.1/partial	425	*MYB*11.m1; organism = *Fragaria* × *ananassa*	gene07416	0.00
*FaMYB1*	FANhyb_icon00014430_a.1.g00001.1/partial	272	*MYB*1.m1; organism = *Fragaria* × *ananassa*	gene09407	1 × 10^−153^
*FaMYB10*	FANhyb_icon00002569_a.1.g00001.1/partial	233	Transcription factor *MYB*113 (At*MYB*113) (simi*LAR* to)	gene31413	1 × 10^−119^
*FaMYB5*	FANhyb_rscf00000101.1.g00008.1	1071	*MYB*5.m1; organism = *Fragaria* × *ananassa*	gene24821	0.00
*FaMYB9*	FANhyb_rscf00002302.1.g00001.1/partial	828	*MYB*9.m1; organism = *Fragaria* × *ananassa*	gene15392	0.00
*FaEOBII*	FANhyb_rscf00000047.1.g00023.1/TE	393	*MYB*-related protein 305 (putative)	gene28435	0.00
***bHLH***					
*FabHLH33*	FANhyb_rscf00000043.1.g00015.1	1962	*BHLH33*.m1; organism = *Fragaria* × *ananassa*	gene19321	0.00
*FabHLH3-delta*	FANhyb_icon00003421_a.1.g00001.1/partial	557	Transcription factor TT8 (*bHLH* 42) (simi*LAR* to)	gene27827	0.00
*FabHLH3*	FANhyb_rscf00003752.1.g00002.1	1041	Transcription factor TT8 (*bHLH* 42) (simi*LAR* to)	gene27827	0.00
*FabHLH1*	FANhyb_icon00000044_a.1.g00001.1	1999	Transcription factor GLABRA 3 (*bHLH* 1) (putative); FaMYC1 mRNA	gene32494	0.00
***WD40***					
*FaWD40-1*	FANhyb_rscf00000569.1.g00002.1	1539	*WD40* repeat-containing protein SMU1 (putative)	gene27104	0.00
*FaWD-1*	FANhyb_icon00020056_a.1.g00001.1/partial	152	WD repeat-containing protein mio (probable)	gene03735	3 × 10^−71^
*FaTTG1*	FANhyb_icon00009619_a.1.g00001.1/partial	367	Protein transparent Testa Glabra 1 (simi*LAR* to); TTG1	gene12450	0.00
*FAWD44-1*	FANhyb_rscf00002089.1.g00001.1	2067	*Fragaria vesca* WD repeat-containing protein 44	gene17869	0.00
**Early Biosynthetic Genes**					
**Phenylalanine**					
*FaPAL1*	FANhyb_rscf00000868.1.g00006.1	1674	Phenylalanine ammonia-lyase 1	gene23261	0.00
*FaPAL2*	FANhyb_rscf00000079.1.g00001.1	2175	Phenylalanine ammonia-lyase 2 (putative)	gene09753	0.00
**Cinnamic Acid**					
*FaC4H*	FANhyb_rscf00000282.1.g00007.1	789	Trans-cinnamate 4-monooxygenase (CA4H) (putative)	gene28093	0.00
*FaCC1*	FANhyb_rscf00000685.1.g00004.1	6963	Biotin carboxylase (probable); Acetyl-CoA carboxylase, AtACC1	gene22077	0.00
**Coumaroyl-CoA**					
*Fa4CL7*	FANhyb_icon00012602_a.1.g00001.1/partial	352	4-coumarate-CoA ligase-like 7 (At4CL6) (putative)	gene09603	0.00
*Fa4CL2*	FANhyb_rscf00001339.1.g00001.1	1644	4-coumarate-CoA ligase 2 (4CL 2) (putative)	gene15877	0.00
**Chalcone**					
*FaCHI3*	FANhyb_icon00000880_a.1.g00001.1/partial	638	Chalcone-flavonone isomerase (probable)	gene21346	0.00
*FaCHI1*	FANhyb_icon00004487_a.1.g00001.1/partial	534	Chalcone-flavonone isomerase 1; TT5	gene23367	0.00
*FaCHS*	FANhyb_icon00003433_a.1.g00001.1/partial	992	Chalcone synthase, TT4; FvCHS	gene26825	0.00
**Flavanone**					
*FaFLS*	FANhyb_icon00020196_a.1.g00001.1/partial	242	Flavonol synthase/flavanone 3-hydroxylase (FLS) (simi*LAR* to)	gene11126	1 × 10^−111^
*FaF3H*	FANhyb_icon00001777_a.1.g00001.1	825	Naringenin,2-oxoglutarate 3-dioxygenase (F3H) (putative)	gene14611	0.00
**Late Biosynthetic Genes**					
**Dihydroflavonol**					
*FaDFR4-1*	FANhyb_rscf00006583.1.g00001.1/partial	578	Bifunctional dihydroflavonol 4-reductase (*DFR*) (probable)	gene29482	1 × 10^−143^
*FaDFR4-2*	FANhyb_rscf00000482.1.g00004.1	2031	Bifunctional dihydroflavonol 4-reductase (*DFR*) (putative)	gene15174	0.00
*FaDFR4-3*	FANhyb_rscf00000482.1.g00003.1	1050	Bifunctional dihydroflavonol 4-reductase (*DFR*) (simi*LAR* to)	gene15176	0.00
**Leucoanthocyanidin**					
*FaLDOX*	FANhyb_icon00007826_a.1.g00001.1/partial	794	Leucoanthocyanidin dioxygenase (*LDOX*) (putative); Fv*LDOX*, TT18	gene32347	0.00
*FaANR*	FANhyb_rscf00000390.1.g00012.1/partial	887	Leucoanthocyanidin reductase (*LAR*) (putative)	gene24665	0.00
*FaLAR*	FANhyb_icon00014507_a.1.g00001.1/partial	497	Leucoanthocyanidin reductase (simi*LAR* to)	gene03877	0.00
**Anthocyanidin**					
*FaUFGT1*	FANhyb_rscf00000061.1.g00006.1	1347	Anthocyanidin 3-*O*-glucosyltransferase 2 (putative)	gene12591	0.00
*FaUFGT2*	FANhyb_rscf00000877.1.g00006.1	1299	Anthocyanidin 5,3-*O*-glucosyltransferase (probable)	gene04355	0.00

## Supplementary Materials

Supplementary Materials can be found at http://www.mdpi.com/1422-0067/19/3/656/s1.

## Abbreviations

F/P	Phenylapropanoid/flavonoid pathway
BGs	Biosynthetic Genes
EBGs	Early Biosynthetic Genes
LBGs	Late Biosynthetic Genes
TF	Transcription Factor
MYB	Myeloblastosis family of transcription factors
bHLH	Basic helix-loop-helix
PAL	Phenylalanine ammonia lyase
C4H	Cinnamate-4-hydroxylase
4CL	4-Coumaroyl-CoA-ligase
CHS	Chalcone synthase
CHI	Chalcone isomerase
F3H	Flavanone 3 hydroxylase
FLS	Flavonol synthase
DFR	Dihydroflavanol reductase
LAR	Leucoanthocyanidin reductase
ANR	Anthocyanidin reductase

## References

[1] Mezzetti B. (2013). Breeding and biotechnology for improving the nutritional quality of strawberry. J. Berry Res..

[2] Schaart J.G., Dubos C., Romero De La Fuente I., van Houwelingen A.M.M.L., de Vos R.C.H., Jonker H.H., Xu W., Routaboul J.M., Lepiniec L., Bovy A.G. (2013). Identification and characterization of *MYB-bHLH-WD40* regulatory complexes controlling proanthocyanidin biosynthesis in strawberry (*Fragaria* × *ananassa*) fruits. New Phytol..

[3] Jaakola L. (2013). New insights into the regulation of anthocyanin biosynthesis in fruits. Trends Plant Sci..

[4] Kayesh E., Shangguan L., Korir N.K., Sun X., Bilkish N., Zhang Y., Han J., Song C., Cheng Z.M., Fang J. (2013). Fruit skin color and the role of anthocyanin. Acta Physiol. Plant..

[5] Tanaka Y., Sasaki N., Ohmiya A. (2008). Biosynthesis of plant pigments: Anthocyanins, betalains and carotenoids. Plant J..

[6] Jimenez-Garcia S.N., Guevara-Gonzalez R.G., Miranda-Lopez R., Feregrino-Perez A.A., Torres-Pacheco I., Vazquez-Cruz M.A. (2013). Functional properties and quality characteristics of bioactive compounds in berries: Biochemistry, biotechnology, and genomics. Food Res. Int..

[7] Lin-Wang K., Liu Y., Espley R.V., Karunairetnam S., McGhie T.K., Hellens R.P., Allan A.C. (2014). Regulation of anthocyanin biosynthesis in strawberry (*Fragaria* sp.) by over-expression of a key transcription factor. Acta Hortic..

[8] Schaefer H.M., Schaefer V., Levey D.J. (2004). How plant–animal interactions signal new insights in communication. Trends Ecol. Evol..

[9] Park K.-I., Ishikawa N., Morita Y., Choi J.-D., Hoshino A., Iida S. (2007). A *bHLH* regulatory gene in the common morning glory, Ipomoea purpurea, controls anthocyanin biosynthesis in flowers, proanthocyanidin and phytomelanin pigmentation in seeds, and seed trichome formation. Plant J..

[10] Pillet J., Yu H., Chambers A.H., Whitaker V.M., Folta K.M. (2015). Identification of candidate flavonoid pathway genes using transcriptome correlation network analysis in ripe strawberry (*Fragaria* × *ananassa*) fruits. J. Exp. Bot..

[11] Koide T., Kamei H., Hashimoto Y., Kojima T., Hasegawa M. (1996). Antitumor effect of hydrolyzed anthocyanin from grape rinds and red rice. Cancer Biother. Radiopharm..

[12] Cristina L., Aizza B., Dornelas M.C., Chen C. (2015). Differential Transcription Factor Networks Orchestrate Flavonoid Biosynthesis. Pigments in Fruits and Vegetables.

[13] Almeida J.R.M., D’Amico E., Preuss A., Carbone F., Deiml B., Mourgues F., Perrotta G., Fischer T.C., Bovy A.G., Martens S., Rosati C. (2007). Characterization of major enzymes and genes involved in flavonoid and proanthocyanidin biosynthesis during fruit development in strawberry (*Fragaria* × *ananassa*). Arch. Biochem. Biophys..

[14] Carbone F., Preuss A., De Vos R.C., Amico E.D., Perrotta G., Bovy A.G., Martens S., Rosati C. (2009). Developmental, genetic and environmental factors affect the expression of flavonoid genes, enzymes and metabolites in strawberry fruits. Plant Cell Environ..

[15] Afrin S., Nuruzzaman M., Zhu J., Luo Z. (2014). Combinatorial interactions of *MYB* and *bHLH* in flavonoid biosynthesis and their function in plants. J. Plant Biol. Res..

[16] Petroni K., Tonelli C. (2011). Recent advances on the regulation of anthocyanin synthesis in reproductive organs. Plant Sci..

[17] Hichri I., Barrieu F., Bogs J., Kappel C., Delrot S., Lauvergeat V. (2011). Recent advances in the transcriptional regulation of the flavonoid biosynthetic pathway. J. Exp. Bot..

[18] Allan A.C., Hellens R.P., Laing W.A. (2008). *MYB* transcription factors that colour our fruit. Trends Plant Sci..

[19] Gonzalez A., Zhao M., Leavitt J.M., Lloyd A.M. (2008). Regulation of the anthocyanin biosynthetic pathway by the TTG1/*bHLH*/*MYB* transcriptional complex in *Arabidopsis* seedlings. Plant J..

[20] Cutanda-Perez M.-C., Ageorges A., Gomez C., Vialet S., Terrier N., Romieu C., Torregrosa L. (2009). Ectopic expression of Vl*MYB*A1 in grapevine activates a narrow set of genes involved in anthocyanin synthesis and transport. Plant Mol. Biol..

[21] Borovsky Y., Oren-Shamir M., Ovadia R., De Jong W., Paran I. (2004). The A locus that controls anthocyanin accumulation in pepper encodes a *MYB* transcription factor homologous to Anthocyanin2 of Petunia. Theor. Appl. Genet..

[22] Grotewold E., Sainz M.B., Tagliani L., Hernandez J.M., Bowen B., Chandler V.L. (2000). Identification of the residues in the *MYB* domain of maize C1 that specify the interaction with the *bHLH* cofactor R. Proc. Natl. Acad. Sci. USA.

[23] Liu Y., Lin-Wang K., Espley R.V., Wang L., Yang H., Yu B., Dare A., Varkonyi-Gasic E., Wang J., Zhang J. (2016). Functional diversification of the potato R2R3 *MYB* anthocyanin activators AN1, *MYB*A1, and *MYB*113 and their interaction with basic helix-loop-helix cofactors. J. Exp. Bot..

[24] Ban Y., Honda C., Hatsuyama Y., Igarashi M., Bessho H., MoriguCHI T. (2007). Isolation and Functional Analysis of a *MYB* Transcription Factor Gene that is a Key Regulator for the Development of Red Coloration in Apple Skin. Plant Cell Physiol..

[25] Espley R.V., Hellens R.P., Putterill J., Stevenson D.E., Kutty-Amma S., Allan A.C. (2007). Red colouration in apple fruit is due to the activity of the *MYB* transcription factor, Md*MYB10*. Plant J..

[26] Jin W., Wang H., Li M., Wang J., Yang Y., Zhang X., Yan G., Zhang H., Liu J., Zhang K. (2016). The R2R3 *MYB* transcription factor Pav*MYB10*.1 involves in anthocyanin biosynthesis and determines fruit skin colour in sweet cherry (*Prunus avium* L.). Plant Biotechnol. J..

[27] Wei H., Chen X., Zong X., Shu H., Gao D. (2015). Comparative Transcriptome Analysis of Genes Involved in Anthocyanin Biosynthesis in the Red and Yellow Fruits of Sweet Cherry (*Prunus avium* L.). PLoS ONE.

[28] Carey C.C., Strahle J.T., Selinger D.A., Chandler V.L. (2004). Mutations in the pale aleurone color1 Regulatory Gene of the Zea mays Anthocyanin Pathway Have Distinct Phenotypes Relative to the Functionally Simi*LAR* TRANSPARENT TESTA GLABRA1 Gene in *Arabidopsis* thaliana. Plant Cell.

[29] Lin-wang K., Bolitho K., Grafton K., Kortstee A., Karunairetnam S., Mcghie T.K., Espley R.V., Hellens R.P., Allan A.C. (2010). An R2R3 *MYB* transcription factor associated with regulation of the anthocyanin biosynthetic pathway in Rosaceae. BMC Plant Biol..

[30] Aharoni A., De Vos C.H.R., Wein M., Sun Z., Greco R., Kroon A., Mol J.N.M., O’Connell A.P. (2001). The strawberry *FaMYB1* transcription factor suppresses anthocyanin and flavonol accumulation in transgenic tobacco. Plant J..

[31] Salvatierra A., Pimentel P., Moya-León M.A., Herrera R. (2013). Increased accumulation of anthocyanins in *Fragaria chiloensis* fruits by transient suppression of *FcMYB1* gene. Phytochemistry.

[32] Lin-Wang K., Micheletti D., Palmer J., Volz R., Lozano L., Espley R., Hellens R.P., Chagne D., Rowan D.D., Troggio M. (2011). High temperature reduces apple fruit colour via modulation of the anthocyanin regulatory complex. Plant. Cell Environ..

[33] Dubos C., Le Gourrierec J., Baudry A., Huep G., Lanet E., Debeaujon I., Routaboul J.-M., Alboresi A., Weisshaar B., Lepiniec L. (2008). *MYB*L2 is a new regulator of flavonoid biosynthesis in *Arabidopsis* thaliana. Plant J..

[34] Miao L., Zhang Y., Yang X., Xiao J., Zhang H., Zhang Z., Wang Y., Jiang G. (2016). Colored light-quality selective plastic films affect anthocyanin content, enzyme activities, and the expression of flavonoid genes in strawberry (*Fragaria* × *ananassa*) fruit. Food Chem..

[35] Rabino I., Mancinelli A.L. (1986). Light, temperature, and anthocyanin production. Plant Physiol..

[36] Takos A.M., Jaffe F.W., Jacob S.R., Bogs J., Robinson S.P., Walker A.R. (2006). Light-Induced Expression of a *MYB* Gene Regulates Anthocyanin Biosynthesis in Red Apples. Plant Physiol..

[37] Medina-Puche L., Cumplido-Laso G., Amil-Ruiz F., Hoffmann T., Ring L., Rodríguez-Franco A., Caballero J.L., Schwab W., Muñoz-Blanco J., Blanco-Portales R. (2014). *MYB10* plays a major role in the regulation of flavonoid/phenylpropanoid metabolism during ripening of *Fragaria* × *ananassa* fruits. J. Exp. Bot..

[38] Lin-Wang K., McGhie T.K., Wang M., Liu Y., Warren B., Storey R., Espley R.V., Allan A.C. (2014). Engineering the anthocyanin regulatory complex of strawberry (*Fragaria vesca*). Front. Plant Sci..

[39] Zhang Y., Li W., Dou Y., Zhang J., Jiang G., Miao L., Han G., Liu Y., Li H., Zhang Z. (2015). Transcript Quantification by RNA-Seq Reveals Differentially Expressed Genes in the Red and Yellow Fruits of *Fragaria vesca*. PLoS ONE.

[40] Fischer T.C., Mirbeth B., Rentsch J., Sutter C., Ring L., Flachowsky H., Habegger R., Hoffmann T., Hanke M.-V., Schwab W. (2014). Premature and ectopic anthocyanin formation by silencing of anthocyanidin reductase in strawberry (*Fragaria* × *ananassa*). New Phytol..

[41] Zhang X., C Allan A., Yi Q., Chen L., Li K., Shu Q., Su J. (2011). Differential Gene Expression Analysis of Yunnan Red Pear, Pyrus Pyrifolia, During Fruit Skin Coloration. Plant Mol. Biol. Report..

[42] Lister C.E., Lancaster J.E., Walker J.R.L. (1996). Developmental Changes in Enzymes of Flavonoid Biosynthesis in the Skins of Red and Green Apple Cultivars. J. Sci. Food Agric..

[43] Zhang H., Yang B., Liu J., Guo D., Hou J., Chen S. (2017). Analysis of structural genes and key transcription factors related to anthocyanin biosynthesis in potato tubers. Sci. Hortic..

[44] Zhang L., Xu B., Wu T., Yang Y., Fan L., Wen M., Sui J. (2017). Transcriptomic profiling of two Pak Choi varieties with contrasting anthocyanin contents provides an insight into structural and regulatory genes in anthocyanin biosynthetic pathway. BMC Genom..

[45] Guo N., Cheng F., Wu J., Liu B., Zheng S., Liang J., Wang X. (2014). Anthocyanin biosynthetic genes in Brassica rapa. BMC Genom..

[46] Livak K.J., Schmittgen T.D. (2001). Analysis of relative gene expression data using real-time quantitative PCR and the 2(*-delta* Delta C(T)) Method. Methods.

[47] Amil-ruiz F., Garrido-Gala J., Blanco-portales R., Folta K.M., Munoz-Blanco J., Caballero J.L. (2013). Identification and Validation of Reference Genes for Transcript Normalization in Strawberry (*Fragaria* × *ananassa)* Defense Responses. PLoS ONE.

