# Supplementary figures and images for: Expression Profiling of Regulatory and Biosynthetic Genes in Contrastingly Anthocyanin Rich Strawberry (Fragaria × ananassa) Cultivars Reveals Key Genetic Determinants of Fruit Color
Source: Int J Mol Sci. 2018 Feb 26;19(3):656. doi: 10.3390/ijms19030656 (PMC5877517; doi:10.3390/ijms19030656)

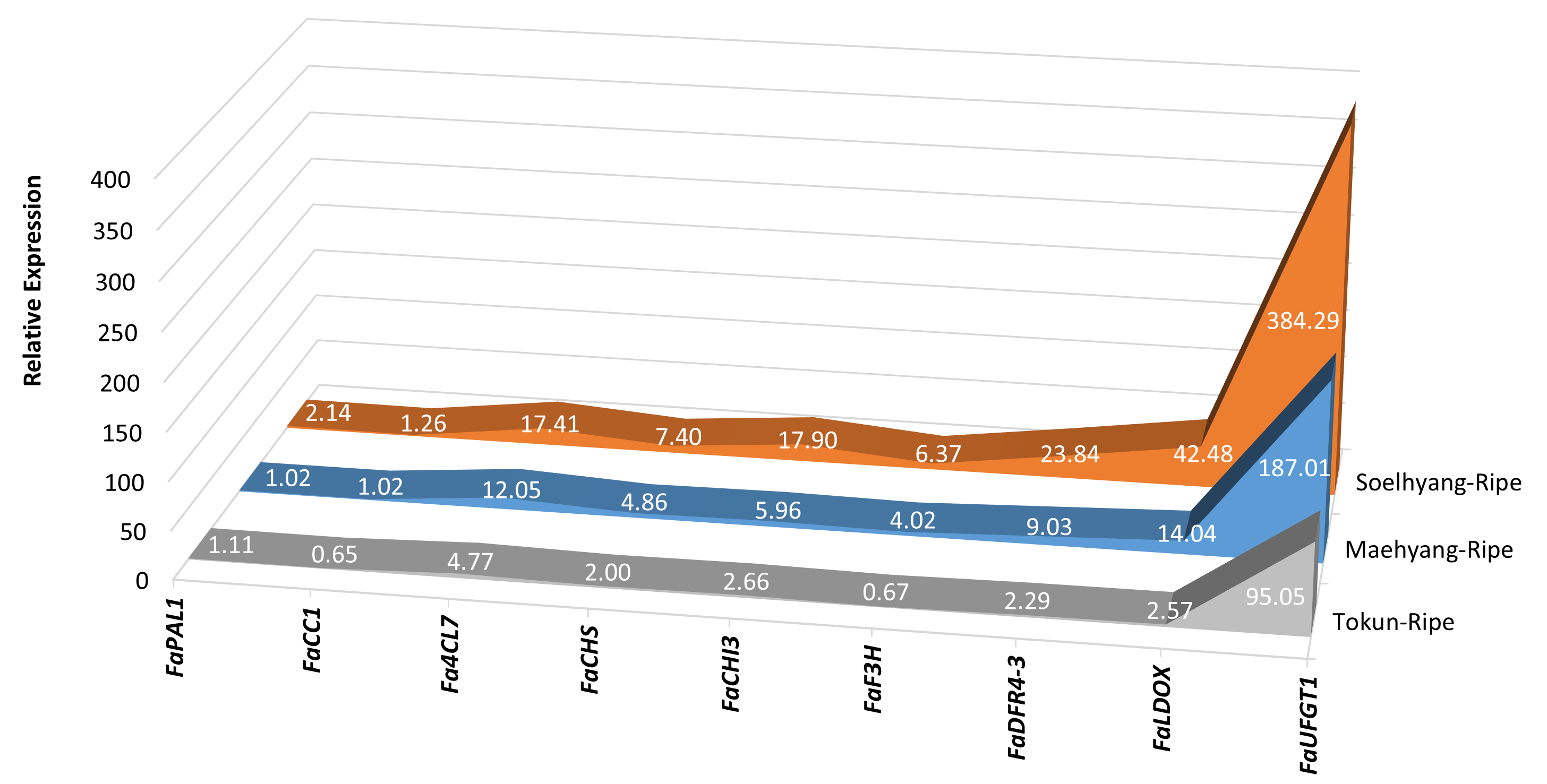

Supplement: Supplementary file 1 [file ijms-19-00656-s001.zip › Supplementary Materials_Hossain etal - IJMS/Figure S1.tif]
